# The Use of Routine Outcome Monitoring in Clinical Supervision: A Scoping Review of Empirical Evidence

**DOI:** 10.1002/cpp.70314

**Published:** 2026-09-09

**Authors:** Arianna Teti, Gianluca Lo Coco, Omar Carlo Gioacchino Gelo, Gloria Lagetto, Giorgio Tasca, Salvatore Gullo

**Affiliations:** ^1^ Department of Psychology, Educational Science and Human Movement University of Palermo Palermo Italy; ^2^ Department of Human and Social Sciences University of Salento Lecce Italy; ^3^ Department of Psychotherapy Sciences Sigmund Freud Private University Vienna Vienna Austria; ^4^ Department of Psychology and Health Sciences Pegaso Telematic University Naples Italy; ^5^ School of Psychology, Faculty of Social Sciences University of Ottawa Ottawa Ontario Canada

**Keywords:** psychotherapy, review, routine outcome monitoring, supervision, training

## Abstract

Routine outcome monitoring (ROM) has been recognized for its potential to enhance treatment outcomes and support decision‐making. This scoping review aimed to systematically map the empirical literature on the use of ROM in clinical and/or training supervision contexts, to summarize the main findings and to identify research gaps. This scoping review also aimed to provide recommendations for future research. Following PRISMA‐ScR guidelines, a systematic search of electronic databases (Scopus, PsycINFO, PubMed, MedLine and Embase) was conducted in March 2025. Studies were included if they reported empirical data on the use of patient‐reported outcome or process measures within supervision. No restrictions were placed on population or methodology. A narrative synthesis was adopted due to heterogeneity among studies. Seventeen studies met the inclusion criteria, varying in methodology, sample and focus. Four key thematic areas emerged: Experiences and Perceived Benefits to Using ROM in Clinical Supervision; Barriers to Using ROM in Clinical Supervision; Effects of ROM on Clinical Outcomes; and Supervisor Training in the use of ROM. While the use of ROM in supervision is promising, current empirical evidence remains fragmented. Key research priorities to inform clinical training and supervisory practice are discussed.

## Summary

- The use of routine outcome monitoring (ROM) within clinical supervision remains infrequent in routine practice, despite theoretical support and emerging empirical interest.
- Discussing patient‐reported outcome data in supervision can provide a structured framework for reflection, support early identification of non‐responding cases and promote more data‐informed clinical decision‐making.
- Supervisor training appears to be a key condition for effective ROM integration, influencing attitudes, confidence and consistency of use in supervision.

## 1 Introduction

Over the past two decades, routine outcome monitoring (ROM) has emerged as a key focus of psychotherapy research. ROM enables clinicians to monitor and enhance patient outcomes and reduce dropout during therapy (Barkham et al. 2023). ROM refers to ‘using information about patient progress and the process of therapy, collected regularly, to guide therapy’ (de De Jong et al. 2023, xi). By providing timely and structured feedback to clinicians and patients (Faija et al. 2022), ROM helps to detect early non‐response and adjust interventions accordingly (Boswell et al. 2015). Its effectiveness in improving clinical outcomes, particularly for off‐track patients, has been demonstrated in several studies and meta‐analyses (e.g., De Jong et al. 2021; Rognstad et al. 2023).

According to the empirical literature and recent theoretical models (e.g., Clinical Performance Feedback Intervention Theory, Brown et al. 2019), the effectiveness of ROM depends not only on the availability of feedback but also on clinicians' ability to interpret and integrate it into their clinical decision‐making, the presence of supportive contextual conditions, adequate training and the opportunity to discuss feedback with knowledgeable supervisors (Brown et al. 2019; De Jong et al. 2021; Herzog et al. 2023).

Despite the growing empirical support, ROM remains underutilized in routine clinical practice (De Jong et al. 2025; Jensen‐Doss et al. 2018). In addition to examining its effect on outcomes, researchers are now exploring its broader impact within the therapeutic process by investigating clinicians' and patients' perceptions (Olive et al. 2025; Låver et al. 2024). Recent studies have shown that ROM enhances the therapeutic alliance, facilitates client verbalization and provides therapists with complementary sources of information to integrate with their clinical judgment (Jonášová et al. 2025). In line with this broader understanding, several additional areas of application and research have emerged, including the use of ROM in clinical supervision and training (McAleavey et al. 2024).

Clinical supervision is a fundamental component of psychotherapist training and ethical clinical practice (Bernard and Goodyear 2014), aiming to foster clinicians' professional development while ensuring the quality of care provided to patients (American Psychological Association 2025; Keum and Wang 2021). While empirical evidence supports that supervision has positive effects on supervisees' specific clinical skills (Keum and Wang 2021), the effect of supervision on client outcome is not clearly established (Klymenko et al. 2025; Schreyer et al. 2025) due to the scarcity of rigorous empirical research (Knox and Hill 2021). Moreover, the methodological weaknesses of most previous studies make it difficult to draw firm conclusions (Borders et al. 2023). Prior research has shown that both therapists and supervisors struggle to identify patients at risk of dropout and tend to overestimate the likelihood of treatment success when relying solely on clinical judgment (Kaiser et al. 2022; Lambert 2010). This issue is particularly relevant given that supervision has traditionally relied on evaluating the supervisee's clinical work through the exchange of subjective impressions and opinions between supervisor and supervisee (Worthen and Lambert 2007).

Taken together, these findings underscore the need for more systematic approaches to integrating patient outcome information into supervision. Incorporating standardized patient feedback into supervision would allow both supervisees and supervisors to complement their clinical judgment with the patient's own perspective on treatment progress and the therapeutic relationship, which is an established predictor of treatment retention and improvement (Bargmann 2017). ROM could help therapists, through collaborative discussion with supervisors, identify areas of their clinical work that may need adjustment and evaluate whether implemented changes lead to improved outcomes. ROM thus may be a valuable tool for assessing which therapist competencies have been consolidated and which require further development (Aafjes‐van Doorn and De Jong 2022).

Despite numerous contributions highlighting the potential benefits of integrating patient‐reported ROM data into clinical supervision, only a few studies have empirically examined its implementation. To the best of our knowledge, no previous work has systematically organized and synthesized the available empirical literature on this topic. The aim of this scoping review was to map the existing empirical literature on the use of patient‐reported ROM data within clinical and/or training supervision contexts, synthesize the available evidence, identify gaps in the current evidence base, and provide recommendations for future research.

## 2 Methods

A systematic literature search was conducted in March 2025 following the Preferred Reporting Items for Systematic Review and Meta‐analysis extension for Scoping Reviews PRISMA‐ScR statement (Moher et al. 2009) across six electronic databases. Reference lists of included studies and grey literature were also searched. Since scoping reviews are designed to analyse highly heterogeneous studies, map the existing literature on a specific topic and identify potential research gaps, this methodology was the most suitable approach for this review.

The complete database‐specific search strategies are reported in Materials S1.

### 2.1 Eligibility Criteria

Studies were eligible if they focused on the use of patient‐generated ROM data—defined as patient‐reported outcome and/or process data collected during therapy—within clinical and/or training supervision contexts. Studies were included if they met the following criteria: (a) reported original empirical data on the use of patient‐generated ROM data within supervision; (b) either directly analyzed patient‐reported ROM data or investigated the use of such data in supervision through empirical methods; (c) included data collected from patients, therapists, supervisors, training directors or multiple stakeholder groups, provided that the focus was on the use of patient‐generated ROM data in supervision; (d) were published in English; (e) involved any type of clinical setting and/or theoretical background of therapists and supervisors; (f) employed any study design.

Studies examining the use of other types of data in supervision (e.g., session video recordings, live supervision) that did not collect patient‐reported data were excluded. Studies were also excluded if ROM was discussed exclusively in relation to therapy processes or clinical outcomes, without any reference to its use within supervision or training contexts. Finally, studies focusing exclusively on professional training in the use of ROM systems or ROM‐related digital platforms were excluded.

### 2.2 Data Extraction

All records identified through the search strategy were independently screened by two authors (AT and GL) based on titles and abstracts. The reviewers compared their screening decisions and discussed any discrepancies. Studies considered potentially eligible by at least one reviewer proceeded to full‐text assessment. Full texts were independently reviewed by the same two authors. Disagreements regarding inclusion or exclusion were discussed during consensus‐oriented meetings until agreement was reached. The entire study selection process was supervised by a senior author (SG).

The following descriptive information was extracted from included studies (if applicable): year of publication, country, study design, study setting, population, sample and aims of the study. To address the research questions, the following information was also extracted from each study: the measure used for ROM, frequency of patient completion, type of therapy, theoretical orientation of the supervisor/trainer and the therapist/trainee, type of supervision, analyses conducted and main findings about the use of ROM in the supervision/training context. To ensure transparency, data extraction was carried out independently by two authors (AT and GL) and supervised through regular meetings by a senior author (SG). Any discrepancies were resolved through discussion aimed at reaching consensus.

Due to the considerable heterogeneity identified among the included studies, a narrative synthesis was adopted to organize the extracted data. Consistent with the aims of a scoping review, no formal quality or risk‐of‐bias assessment of the included studies was conducted. Given the substantial heterogeneity in study designs, populations and outcomes, a formal risk of bias assessment was beyond the scope of the present review. An analytical framework was developed to synthesize the findings. Following data extraction, study aims, outcomes and key findings were reviewed and compared across studies. An inductive thematic grouping approach was adopted to organize the evidence. Two authors (AT and GL) independently examined the extracted findings and identified recurring topics across studies, which were grouped into preliminary thematic categories. A third independent author (GT) reviewed the thematic structure and refined the categories to support credibility. These categories were subsequently refined through discussion and consensus, resulting in the four final thematic areas presented in Section 3.

## 3 Results

### 3.1 Systematic Search Results

**FIGURE 1:**
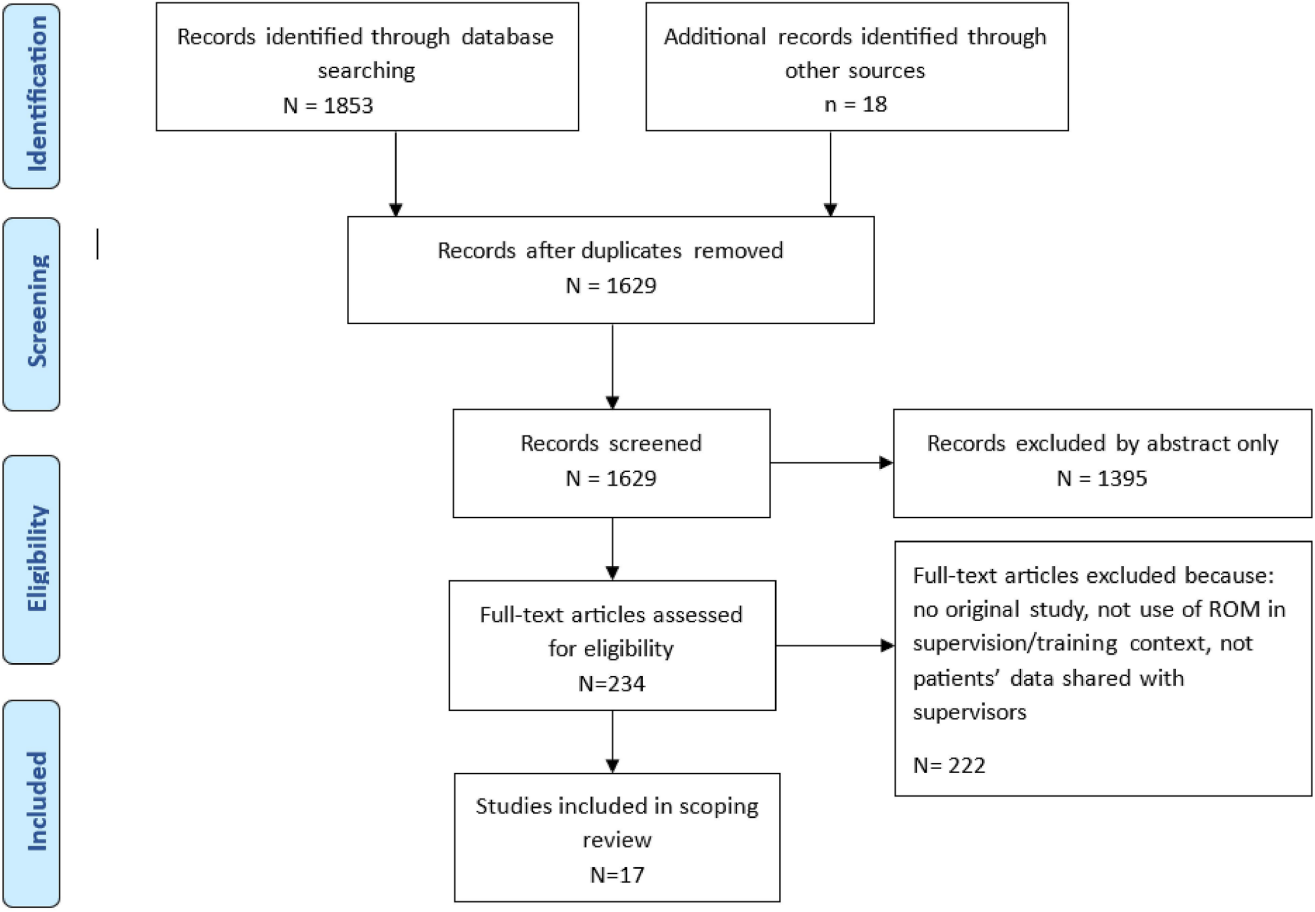
Flowchart of studies included in the present scoping review.

A total of 1853 articles were identified through database searches, in addition to 18 articles retrieved through other sources. After screening, 234 full‐text articles were assessed for eligibility, and 17 studies were included in this scoping review. A flow‐chart of study selection is presented in Figure 1.

### 3.2 Study Characteristics

The 17 studies included in this review were conducted between 2009 and 2024. These studies were conducted primarily in the United States (*k* = 10) with quantitative (*k* = 9), qualitative (*k* = 4) and mixed‐methods (*k* = 4) designs. Samples varied widely and included patients, therapists, supervisors, trainees and training directors. The studies' characteristics are reported in Table 1.

**TABLE 1:** Characteristics of included studies.

Author(s)	Country	Publication type	Research design	Sample size	ROM measures
Axelsson et al. (2023)	Sweden	Article	Qualitative	T = 6; S = 2	ORS, SRS, LASS
Dastrup (2022)	USA	Thesis	Longitudinal	S = 52	N.S.
Davidson et al. (2017)	Scotland	Article	RCT	125; T = 19; S = 19	CORE‐10, CGI
Dexter (2017)	USA	Thesis	Mixed‐methods	T = 318	N.S.
Fullerton et al. (2018)	England	Article	Mixed‐methods	S = 42	N.S.
Goldberg et al. (2016)	USA	Article	Longitudinal	5128; T = 153	OQ‐45
Grossl et al. (2014)	USA	Article	RCT	138; T = 44, S = 18	ORS, SRS
Minieri et al. (2015)	USA	Article	Case‐report	T = 3	ORS, SRS
Overington et al. (2016)	Canada	Article	Cross‐sectional	TD = 238	N.S.
Rast (2018)	USA	Thesis	Mixed‐methods	S = 181	N.S.
Reese et al. (2009)	USA	Article	RCT	110; T = 28; S = 9	ORS, SRS
Rosén (2019)	Sweden	Thesis	Longitudinal	12; T = 12; S = 2	ORS, SRS, LASS
Rosval et al. (2019)	Canada	Article	Mixed‐methods	24; T = 49; S = 17	OQ‐45, YOQ
Rousmaniere, Swift, et al. (2014)	USA	Article	Retrospective	C = 6562; T = 175; S = 23	OQ‐45
Sparks et al. (2011)	USA	Article	Case‐report	T = N/A	ORS, CORS, SRS, CSRS
Valdiviezo‐Oña et al. (2024)	Ecuador	Article	Qualitative	S = 2	N.S.
Whipple et al. (2020)	USA	Article	Retrospective	3300; T = 80; S = 39	OQ‐45

Abbreviations: CGI = Clinical Global Impression; CORS = Child Outcome Rating Scale; CSRS = Child Session Rating Scale; LASS = Leeds Alliance in Supervision Scale; N.S. = not specified; OQ‐45 = Outcome Questionnaire‐45; ORS = Outcome Rating Scale; P = patients; S = supervisors; SRS = Session Rating Scale; T = therapists; TD = training directors; YOQ = Youth Outcome Questionnaire.

Although not all included studies collected empirical data directly from patients, all studies focused on the use of patient‐generated ROM data within supervision contexts. Some studies examined the use of such data through reports provided by therapists, supervisors, trainees or training directors rather than through direct analyses of patient data. Although the demographic and clinical information regarding therapists, supervisors (such as age, gender and theoretical orientation) and patient characteristics, as well as some details of the supervision and treatment delivered (e.g., duration, therapeutic model, setting), were initially included in the data extraction matrix, these variables were inconsistently reported across studies, were highly heterogeneous and underspecified. As a result, they are not presented in Section 3, as they could not be meaningfully summarized or synthesized.

### 3.3 Measures

All studies used session‐by‐session patient‐reported outcome measures, although the instruments varied across studies. The Outcome Rating Scale (ORS) and Session Rating Scale (SRS; Miller et al. 2015) and the Outcome Questionnaire‐45 (OQ‐45; Lambert et al. 2004) were the most commonly used measures. A small number of studies employed other outcome, alliance or clinician‐rated measures (see Table 1).

### 3.4 Research Questions and Study Aims

The studies included in this scoping review were characterized by an explicit focus on the use of ROM data collected from patients in the supervision of both licensed clinicians and trainees. However, studies differed in their specific aims and the perspectives adopted to investigate the role of ROM in supervision. Some studies (*k* = 6, 35.29%) examined how ROM was incorporated into supervision practice, exploring the experiences and perspectives of the different parties involved in the process. Four of these studies examined the experiences of supervisees (Axelsson et al. 2023; Minieri et al. 2015; Sparks et al. 2011; Dexter 2017), one investigated the perspective of supervisors (Valdiviezo‐Oña et al. 2024) and one adopted a multi‐perspective approach of supervisees, supervisors and patients (Rosval et al. 2019).

Another subgroup of studies (*k* = 5, 29.41%) evaluated the effectiveness of ROM implementation in supervision across multiple outcomes, with some studies contributing to more than one outcome category. These included therapist skills (e.g., self‐compassion; Reese et al. 2009; Rosén 2019) and supervisees' satisfaction and perceived supervisory alliance (Grossl et al. 2014; Reese et al. 2009). Four studies evaluated patient treatment outcomes as indicators of the effectiveness of using ROM within supervisory settings (Davidson et al. 2017; Goldberg et al. 2016; Grossl et al. 2014; Reese et al. 2009).

Regarding the remaining studies, two aimed to examine the extent to which patient outcome variability was explained by supervisors when supervision was informed by ROM data (Rousmaniere, Swift, et al. 2014; Whipple et al. 2020). Two studies evaluated the efficacy of a training programme to optimize supervisors' utilization of ROM in clinical supervision (Dastrup 2022; Fullerton et al. 2018). Finally, two survey studies examined the use of ROM data in clinical supervision from the perspectives of training centre directors (Overington et al. 2016) and clinical supervisors (Rast 2018). A summary of the specific aims and key findings of each included study is provided in Table 2.

**TABLE 2:** Study aims and main findings of included studies.

Author(s)	Study aims	Main findings
Axelsson et al. (2023)	To explore supervisees' experiences with ROM in supervision, including perceived utility and barriers.	ROM supported structured reflection, supervision process and clinical insight. Barriers included time constraints, unclear guidelines and lack of training.
Dastrup (2022)	To evaluate the effects of a supervisor training program on ROM use.	Training improved supervisors' attitudes, self‐efficacy and consistency in using ROM during supervision.
Davidson et al. (2017)	To assess whether sharing ROM data with supervisors affects treatment outcomes and supervision process.	No significant outcome differences: however, ROM‐informed therapies were shorter. Therapists not sharing ROM perceived greater client improvement.
Dexter (2017)	To examine clinicians' use of ROM in supervision and its perceived value.	ROM was rarely used; those using it reported more frequent supervision. ROM aided treatment redirection and competency development.
Fullerton et al. (2018)	To assess the impact of supervisor training on ROM use.	Training led to improved supervisor attitudes, self‐efficacy and perceived ability to guide therapists. Increased ROM use was confirmed by both supervisors and supervisees.
Goldberg et al. (2016)	To examine the effect of ROM feedback on treatment effectiveness.	Implementation of ROM with expert discussion improved treatment outcomes.
Grossl et al. (2014)	To evaluate ROM's impact on supervisee satisfaction and alliance.	No difference in supervisory alliance; supervisees who discussed ROM reported higher satisfaction.
Minieri et al. (2015)	To investigate supervisees' perceptions of ROM in supervision.	ROM helped incorporate client voice, structure supervision and foster openness in discussing challenges.
Overington et al. (2016)	To explore ROM use in supervision across training clinics.	Only 26.5% used ROM. Barriers included time demands, clinician resistance and concerns about patient reactions.
Rast (2018)	To examine supervisor attitudes and behaviours regarding ROM.	Supervisors preferred clinical judgement over ROM. Barriers included lack of training and guidance.
Reese et al. (2009)	To assess ROM's impact on outcomes, self‐efficacy, alliance and satisfaction.	Better outcomes and stronger self‐efficacy–outcome link were found in the ROM group. No differences in alliance or satisfaction.
Rosén (2019)	To examine ROM's role in enhancing clinicians' self‐compassion and its relation to outcomes.	ROM use increased supervisees' self‐compassion, which was associated with better patient outcomes. No effects for supervisors.
Rosval et al. (2019)	To investigate ROM use in supervision from multiple perspectives.	ROM use was limited and trainee initiated. Barriers included perceived irrelevance, time issues and limited training.
Rousmaniere, Swift, et al. (2014)	To examine supervisor contributions to outcomes in ROM‐informed therapy.	Supervisors explained 0% of the variance in patient outcomes.
Sparks et al. (2011)	To explore how client feedback is used in supervision.	ROM enhanced learning, clinical structure and progress monitoring.
Valdiviezo‐Oña et al. (2024)	To investigate supervisors' perceptions of ROM use in supervision.	ROM supported clinical reasoning and process improvement. Barriers included time, technical issues and insufficient training.
Whipple et al. (2020)	To estimate outcome variance explained by supervisors in ROM‐informed therapy.	Supervisors accounted for only 2% of the variance in patient outcomes.

### 3.5 Main Outcomes

Consistent with the aim of mapping the empirical literature on ROM in supervision, the included studies examined different applications of patient‐reported ROM data within supervisory contexts, reflecting the conceptual and methodological diversity of this emerging field. Table 2 provides a detailed overview of the objectives and principal findings of each included study. The main results reported across these studies were organized into four thematic areas: (1) Experiences and Perceived Benefits of ROM in Clinical Supervision, (2) Barriers to the use of ROM in Clinical Supervision, (3) Supervisor Training on the use of ROM and (4) Effects of ROM on Supervision and Clinical Outcomes. We note instances in which studies reported findings pertinent to multiple thematic domains and provide a comprehensive summary of the findings in the respective sections.

#### 3.5.1 Experiences and Perceived Benefits of ROM in Clinical Supervision

Eight studies contributed to this thematic area on findings related to the experiences of patients, clinicians and supervisors regarding the use and perceived usefulness of ROM within the context of clinical supervision. This theme includes studies that examined the use of patient‐reported ROM data (i.e., outcome and process measures collected during therapy) within supervision. These studies include both routine clinical supervision contexts and supervision models explicitly structured to integrate ROM data into supervisory practice. Regarding studies examining the use of ROM data in routine clinical practice, Dexter (2017) found that only 19.3% of clinicians discussed patient‐reported ROM data during supervision sessions. Those who did so also reported receiving more frequent supervision. Similar findings were reported by Overington et al. (2016), who found that only 26.5% of training clinic directors reported collecting and discussing patient‐reported ROM data during supervision. Rast (2018) found that most supervisors in their sample preferred to rely on their clinical judgement when conducting supervision rather than incorporating patient‐reported ROM data. In line with these findings, Rosval et al. (2019) described limited integration of patient‐reported ROM data into supervision. Discussions of ROM data were rare, often initiated by trainees, and influenced by supervisors' level of familiarity with and interest in these tools. Regarding research investigating therapists' experiences with a supervision model based on ROM data, all studies reported that participants recognized the potential of using patient‐reported ROM data in supervision to enhance treatment outcomes. Discussing ROM data within supervisory contexts was considered helpful for introducing the patient's perspective into the conversation (Minieri et al. 2015; Overington et al. 2016; Sparks et al. 2011), providing a structure and framework for the supervision process and connecting quantitative indicators to clinical insight and judgement (Axelsson et al. 2023; Rosval et al. 2019; Sparks et al. 2011; Valdiviezo‐Oña et al. 2024). These studies also described tracking of patient progress throughout the course of therapy and identifying when an intervention may need to be redefined or redirected as useful (Dexter 2017; Minieri et al. 2015; Rosval et al. 2019; Sparks et al. 2011; Valdiviezo‐Oña et al. 2024). Also helpful was highlighting of specific clinical competencies or skill domains that trainees needed to consolidate or strengthen (Axelsson et al. 2023; Sparks et al. 2011; Valdiviezo‐Oña et al. 2024). Finally, discussion of patient‐reported ROM data during supervision was perceived as a beneficial tool for supporting and strengthening the supervisory relationship itself (Axelsson et al. 2023; Minieri et al. 2015), fostering a less defensive attitude among clinicians when confronting weaker aspects of their clinical work (Minieri et al. 2015).

#### 3.5.2 Barriers to the Use of ROM in Clinical Supervision

Six studies contributed to this thematic area. They highlight several barriers to the effective use and discussion of ROM within supervision. First, time‐ and cost‐related barriers during therapy (in relation to data collection) and during supervision (in relation to data review) were reported, which affected the integration of ROM systems into clinical practice (Axelsson et al. 2023; Dexter 2017; Overington et al. 2016; Rosval et al. 2019). Second, technical difficulties were also mentioned as an obstacle to ROM use (Valdiviezo‐Oña et al. 2024). A third barrier was related to clinicians' resistance to collecting ROM data (Overington et al. 2016). This resistance was partly linked to clinicians' concerns that patients might react negatively or that the process could interfere with the therapeutic relationship (Rosval et al. 2019; Valdiviezo‐Oña et al. 2024). A fourth commonly reported barrier was that the instruments used were not always viewed as relevant or suitable for working with certain patients (Rosval et al. 2019).

A fifth commonly reported barrier was insufficient training for both therapists and supervisors, which limited their ability to effectively integrate ROM data into supervision. This led to difficulties in understanding how to read and interpret the ROM data, thereby reducing its perceived clinical utility (Axelsson et al. 2023; Valdiviezo‐Oña et al. 2024). Finally, the absence of clear guidelines and indications was associated with uncertainty about how to introduce and discuss ROM not only within supervision but also with patients (Axelsson et al. 2023; Dexter 2017; Rast 2018; Rosval et al. 2019). As a consequence, ROM data were often perceived by therapists as external to the therapeutic process, reducing their motivation to use them consistently (Axelsson et al. 2023).

#### 3.5.3 Supervisor Training on the Use of ROM

Among the studies included in this review, two papers specifically examined the effects of training programs aimed at enhancing supervisors' ability to integrate ROM data into clinical supervision and reported comparable results. Both studies found that, after the training, supervisors reported a more positive attitude towards using ROM in supervision, along with increased self‐efficacy and a greater perceived ability to teach supervisees how to use and interpret ROM data. After training, supervisors from both samples used the ROM data more consistently during supervision, and this change was also confirmed by the supervisees (Fullerton et al. 2018).

#### 3.5.4 Effects of ROM on Supervision and Clinical Outcomes

Seven studies examined how the use of patient‐reported ROM data within the supervision session may influence treatment outcomes, the professional development of trainees and some process‐related aspects of supervision.

Goldberg et al. (2016) reported improved treatment effectiveness following the implementation of a ROM system within an agency. The ROM system consisted of routine outcome data collection and regular discussions of feedback with expert consultants. Similarly, Reese et al. (2009) reported better outcomes among therapists who discussed ROM data with their supervisors than among those who did not. Rosén (2019) documented a significant increase in clinicians' self‐compassion over the course of supervision that incorporated ROM data. Supervisees' self‐compassion levels were positively correlated with patient outcomes, while no such association was observed for supervisors' self‐compassion. Davidson et al. (2017) and Grossl et al. (2014) found no statistically significant differences in clinical outcomes between patients whose ROM data were shared with supervisors and those whose data were not. However, Davidson et al. (2017) found that therapies in which ROM data were reviewed during supervision were, on average, shorter. Notably, therapists in the condition where ROM data were not shared with supervisors rated their patients as having improved more than those in the data‐sharing condition. Finally, Rousmaniere, Swift, et al. (2014) and Whipple et al. (2020) found that in therapies where ROM data were integrated into supervision, differences between supervisors accounted for a very small proportion of variability in patient outcomes (0% and 2% of the variance, respectively). This finding suggests limited supervisor‐level effects on outcome variability.

Focusing on the development of professional skills, Reese et al. (2009) found that the correlation between therapists' self‐efficacy and patient outcomes was stronger among therapists who discussed ROM data in supervision compared to those who did not. The authors suggested that feedback in supervision may help clinicians develop a more accurate perception of their performance.

Regarding the perceived supervisory alliance, Reese et al. (2009) and Grossl et al. (2014) reported no significant differences between therapists who discussed ROM data and those who did not. In contrast, the results of these two studies differed in terms of trainees' satisfaction with supervision. Grossl et al. (2014) found higher levels of satisfaction among trainees who shared ROM data with supervisors, whereas the Reese et al. (2009) study did not detect any significant differences.

## 4 Discussion

The aim of this scoping review was to map the empirical literature on the use of ROM in clinical supervision, with a specific focus on the manner in which supervisors and therapists employ patient‐reported ROM data within supervisory contexts. Applications of ROM aimed at monitoring supervision processes, supervisory relationships or supervision outcomes were considered conceptually distinct and therefore fell outside the scope of this review. One of the main findings of the review was that patient‐reported ROM data can serve multiple functions within supervisory contexts, including supporting clinical decision‐making, therapist development and supervisory discussions. The broad range of applications highlights the extensive potential of ROM in the context of supervision, while also contributing to the heterogeneity of empirical findings. The findings were organized into four thematic areas: (1) Experiences and Perceived Benefits of ROM in Clinical Supervision; (2) Barriers to the use of ROM in Clinical Supervision; (3) Supervisor Training in the use of ROM; and (4) Effects of ROM on Supervision and Clinical Outcomes.

The first thematic category yielded the largest number of findings, suggesting that both therapists and supervisors acknowledge the potential benefits of ROM. Its primary value lies in the opportunity to structure and enrich supervision through a quantitative assessment from the patient's perspective, thereby complementing clinical judgement (Rousmaniere, Abbass, and Frederickson 2014). However, the studies highlighted that the use of ROM data in supervision remains limited in practice, an issue that extends beyond the supervision context (e.g., De Jong et al. 2025). According to the second thematic area, several barriers hinder ROM's effective implementation, particularly the lack of adequate training for supervisors on integrating ROM data into the supervision. Prior studies found that lack of training is one of the main reasons clinicians choose not to use ROM with their patients (Cook et al. 2017; De Jong et al. 2021). Furthermore, the lack of guidance on integrating ROM into therapy may cause clinicians to worry that it could harm the therapeutic relationship (Valdiviezo‐Oña et al. 2024). However, training can improve clinicians' attitudes towards ROM (Waldron et al. 2018). The findings of the present review suggest that supervisor training increases supervisors' confidence in using ROM. This enhances their ability to support supervisees in interpreting feedback by developing supervisors' competence to integrate ROM effectively into supervision.

Regarding the fourth thematic area, our results show that the existing literature on the effects of ROM on supervision and clinical outcomes is still limited and fragmented. The current body of evidence does not yet support definitive conclusions. While some studies suggest a potential effect of ROM on improving patient outcomes, the only existing randomized controlled trial did not find significant effects (Davidson et al. 2017). However, the same trial reported shorter treatment duration when ROM data were shared with supervisors, despite comparable patient outcomes. Clinicians rated treatments as more effective when supervisors did not receive ROM data, a finding that should be interpreted in light of clinicians' tendency to overestimate treatment effectiveness (Walfish et al. 2012). Similarly, studies incorporating ROM data into supervision reported that supervisors accounted for only a limited proportion of the variance in patient outcomes. However, these findings require further confirmation and clarification, as they may indicate either that regular ROM feedback reduces differences between supervisors by providing a shared data‐driven framework, or that supervisor feedback has limited impact. Future research should examine how and to what extent supervisors use feedback (e.g., Hannan et al. 2005; Lambert et al. 2018).

Some studies also reported associations between ROM use in supervision and the development of therapist capacities, such as self‐efficacy, that could be linked to more effective clinical outcomes (Clements‐Hickman and Reese 2023; Janse et al. 2023). However, given the exploratory nature of these studies and the lack of control for confounding factors, their findings should be interpreted with caution.

Finally, when considering the impact of ROM on the supervision process, the limited available evidence does not allow one to determine whether ROM meaningfully influences the relational dynamics between supervisor and supervisee. Consequently, this remains an open question for future research.

Beyond summarizing existing findings, a key objective of this scoping review was to identify gaps in the current literature. To date, only a limited number of studies have investigated the use of ROM in clinical supervision. Nevertheless, most of the included studies were conducted in the past decade, suggesting growing scientific interest in this application of ROM (Aafjes‐van Doorn and De Jong 2022). Current evidence remains concentrated in a few countries and has focused primarily on trainee therapists, highlighting the need for broader investigation across clinical settings and professional populations (Rousmaniere, Abbass, and Frederickson 2014). The 17 studies in this review encompassed a broad range of methodologies, ranging from RCT to case reports and mixed‐methods designs. Although the heterogeneity of study aims offers valuable insights into how ROM may influence various levels of clinical supervision, this variability limits the ability to draw firm conclusions about its specific impact.

Several important areas appear to remain unexplored or insufficiently addressed, warranting further empirical investigation. Most studies provided limited contextual information (e.g., characteristics of supervisors, therapists, patients or the nature of treatment), making it difficult to determine under which conditions ROM may be most effective in supervision. In addition, in most of the included studies, the specific procedures for integrating ROM data into supervision sessions were not clearly described. This lack of detail limits the ability to identify a replicable model that could be implemented in routine clinical practice. A more general methodological limitation is the absence of control variables or control groups. This limits the ability to draw valid conclusions about the impact of ROM‐informed supervision on therapy outcomes. Moreover, most studies focused on patient outcomes rather than therapeutic process variables, even though the value of monitoring process variables alongside outcomes is well documented (Boswell 2020). Finally, the reliance on small and context‐specific samples limits the generalizability of the findings. Overall, although preliminary evidence suggests potential benefits of integrating ROM into supervision, this area of research remains in its early stages and requires further empirical development.

This review has several limitations. First, consistent with scoping review methodology, no formal quality assessment of the included studies was conducted. As a result, we did not evaluate the methodological rigour or risk of bias in the studies, which limited our ability to critically appraise the reported findings. Second, the wide range of studies included made it difficult to make direct comparisons and draw overarching conclusions. Third, the search was restricted to English‐language publications, and despite efforts to include grey literature, some relevant unpublished studies may have been missed. Finally, incomplete reporting in several studies, such as characteristics of supervisees, supervisors, patients or therapeutic models, limited the synthesis of important contextual information, reducing the depth and generalizability of the findings.

## 5 Future Research Areas and Research Priorities

Despite promising preliminary findings, the empirical literature on the use of ROM within clinical supervision remains scarce and fragmented. To generate evidence that meaningfully informs clinical training and supervisory practice, future research should address several key priorities.

Randomized controlled trials with adequate control conditions are needed to evaluate whether systematic use of ROM within supervision and supervisor training in the use of ROM contributes to improvements in patient outcomes, therapists' professional development and supervision processes. Studies should account for the nested structure of the data and explicitly model its multilevel nature (e.g., patients nested within therapists, and therapists nested within supervisors). Analyses should distinguish between‐ and within‐supervisor and therapist effects to separate supervisor‐level characteristics from therapist‐ and patient‐level variability. Such designs would allow researchers to simultaneously examine effects at the supervisor, therapist, and patient effects, as well as potential cross‐level interactions between supervisor training and patient‐level ROM use.

Future research should provide more detailed and transparent information about the supervisory conditions under which ROM is implemented. This could include aspects such as supervision models, theoretical orientation, supervision frequency and structure, and the specific ways in which ROM data are discussed during supervision sessions.

Future studies should explore the potential impact of integrating ROM process measures on the supervisory relationship. Exploratory longitudinal studies in which patients complete not only outcome measures but also process measures (e.g., therapeutic alliance) may provide valuable insights into how supervisors use relational data to inform supervision and support clinical decision‐making. Although beyond the scope of the present review, preliminary evidence suggests that feedback‐informed approaches may also have potential applications for monitoring supervision‐specific processes and outcomes, such as supervisory alliance (Worthen and Lambert 2007), supervisees' learning goals (Valdiviezo‐Oña et al. 2022) and supervision effectiveness (Worthen and Lambert 2007). Research is needed to systematically map this emerging body of literature and to better understand whether the mechanisms underlying ROM can support the supervisory process.

Finally, a deeper understanding of the attitudinal and contextual factors influencing ROM implementation in supervision is essential. Qualitative studies (Gelo et al. 2020) could examine therapists' and supervisors' attitudes towards ROM, how monitoring data are used in supervision, how feedback is interpreted and negotiated within supervisory relationships and how ROM data are integrated with clinical judgment.

Taken together, these research priorities highlight the need to develop shared, evidence‐informed guidelines for the use of ROM in clinical supervision. The findings of this review indicate that, in the absence of clear guidance, ROM integration into supervision remains inconsistent and largely dependent on individual supervisor preferences rather than on empirically grounded principles. Future research should aim to develop flexible guideline frameworks that specify key components of ROM‐informed supervision, such as which types of data are collected, when and how outcome information is introduced and how ROM data are integrated with supervisory goals and clinical judgment. Overall, advancing this coordinated, methodologically diverse research agenda, summarized in Table 3, will be essential for translating preliminary evidence into best practices for ROM‐informed clinical supervision.

**TABLE 3:** Research gaps and priorities in ROM‐informed clinical supervision.

Identified gap in the current literature	Research priority	Recommended study design	Key outcomes
Limited causal evidence on the effectiveness of ROM use in supervision and supervisor training in ROM	Clarify the effects of ROM‐informed supervision and supervisor training on clinical and supervisory outcomes	Multilevel randomized controlled trials with supervisors randomized to ROM training vs. no training, and patients randomized to ROM vs. no‐ROM conditions	Patient clinical outcomes; therapist professional development; supervision processes
Lack of information of supervisory conditions in ROM‐informed supervision	Identify supervisory contexts and conditions under which ROM is implemented and potentially effective	Cross‐sectional studies and structured surveys of supervision practices	Supervision models, theoretical orientation, frequency and structure of supervision and concrete modalities of ROM data integration within supervision
Limited investigation of ROM process measures within supervision contexts	Examine the role of ROM process measures in informing supervision and supervisory relationships	Longitudinal studies incorporating both outcome and process measures	Therapeutic alliance, supervisory alliance and use of relational data in supervisory decision‐making
Underdeveloped qualitative evidence on ROM‐informed supervision	Explore supervisors' and therapists' experiences using ROM through multi‐source qualitative data	Qualitative studies integrating multiple data sources (e.g., interviews, focus groups, and recordings of supervision sessions) using rigorous analytic frameworks	Meaning‐making processes, negotiation of feedback, and integration of ROM data with clinical judgement
Absence of shared, evidence‐informed guidance on how to use ROM in supervision	Develop flexible, evidence‐informed guidelines for ROM integration in supervision	Mixed‐methods studies and consensus‐building approaches	Feasibility and clinical applicability of guideline frameworks

In conclusion, although research on the use of patient‐reported ROM data in clinical supervision is still in its early stages, current evidence suggests that it represents a promising approach to enhancing therapist development and improving patient care. The present scoping review focused specifically on the use of patient‐generated treatment data in supervision and identified key research priorities to advance the empirical evidence base, inform best practices, and support the effective implementation of ROM in supervision.

## Funding

The authors have nothing to report.

## Disclosure

No previously published material requiring permission has been reproduced in this article.

## Ethics Statement

The authors have nothing to report.

## Consent

The authors have nothing to report.

## Conflicts of Interest

The authors declare no conflicts of interest.

## Supporting information


**Data S1:** Supporting information.

## Data Availability

This study is a scoping review of previously published literature. No new data were created or analysed.
