# Supplementary material for: The Use of Routine Outcome Monitoring in Clinical Supervision: A Scoping Review of Empirical Evidence
Source: Clin Psychol Psychother. 2026 Sep 9;33(5):e70314. doi: 10.1002/cpp.70314 (PMC13555173; doi:10.1002/cpp.70314)
Supplement: Supplementary file 1 [file CPP-33-e70314-s001.docx]

**Supplementary Material S1. Search strategy used for the literature search.**

The literature search was conducted using the following electronic databases: Scopus, PsycINFO, PubMed, MedLine, Embase and ProQuest. The search was limited to articles published between 2001 and March 2025. The search was conducted using three main groups of concepts and their synonyms: (a) supervision OR training (supervis*, train*, student*); (b) psychotherapy (psychotherap*, therap*, counsel*, clinical assessment*); and (c) routine outcome monitoring ("ROM", "feedback-informed treatment*", "FIT", "routine outcome measurement", "progress feedback", "outcome assessment*", "outcome monitor*"). The search was performed on titles, keywords, and abstracts, using the "AND" operator to connect the three categories of terms (a, b, and c). The reference lists of the studies included in the scoping review were manually screened to identify any additional relevant studies. Finally, as we were also interested in grey literature, a call for the submission of unpublished works was distributed via email through scientific associations and listservs.
